# Response of the German public health service to the first imported mpox clade Ib case in Germany, October 2024

**DOI:** 10.2807/1560-7917.ES.2024.29.28.2400743

**Published:** 2024-11-28

**Authors:** Rosanne de Jong, Jennifer Schauer, Annelene Kossow, Sibylle Scharkus, Annette Jurke

**Affiliations:** 1 Infectious Disease Epidemiology, North Rhine-Westphalia Centre for Health (LZG.NRW), Bochum, Germany; 2 ECDC Fellowship Programme, Field Epidemiology path (EPIET), European Centre for Disease Prevention and Control (ECDC), Stockholm, Sweden; 3 Local health department of Cologne, Cologne, Germany; 4 Institute of Hygiene, University Hospital Muenster, University of Muenster, Muenster, Germany; * These authors contributed equally to this work and share first authorship.

**Keywords:** Mpox, Monkeypox virus, Communicable Diseases, Imported, Contact Tracing, Case Management

## Abstract

In October 2024, the first imported mpox clade Ib case was confirmed in Germany in an individual in their thirties returning from Rwanda. In this report we summarise the response from the public health service in North Rhine-Westphalia related to case management, contact tracing and institutional collaborations. Our findings highlight the importance of a coordinated public health response in the management of imported mpox cases and in preventing the transmission of mpox clade Ib in Germany and beyond.

In June 2024, the World Health Organization (WHO) reported a new sub-lineage of clade I monkeypox virus (MPXV), named clade Ib, which is estimated to have emerged in mid-September 2023 in the Democratic Republic of the Congo (DRC) and has since spread to Burundi, Kenya, Rwanda, Uganda, Zambia and Zimbabwe [1,2].

Here we report on the coordinated public health response to the first confirmed MPXV clade Ib infection imported into Germany in a traveller returning from Rwanda in October 2024.

## Case description

**Figure 1:**
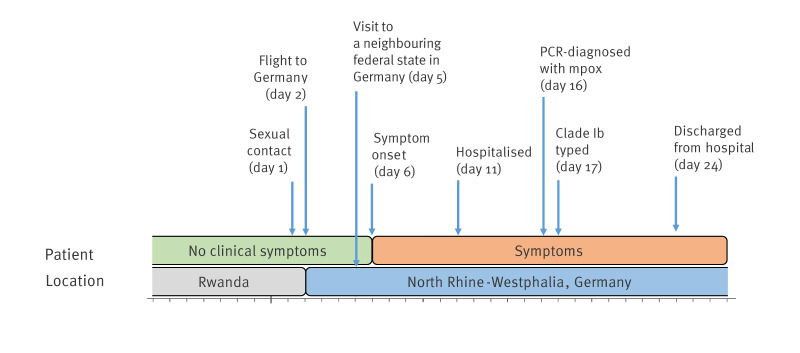
Timeline of disease onset and travel history of the first mpox clade Ib case detected in Germany, September–October 2024

In mid-October 2024, the local health department of Cologne notified the North Rhine-Westphalia federal state public health institute (LZG.NRW) of a PCR-confirmed mpox diagnosis in an individual aged in their thirties. The next day, the conciliary laboratory for smallpox viruses at the Robert Koch Institute (RKI), which is the national public health institute in Germany, confirmed the infection as MPXV clade Ib by molecular typing [3]. Patient history revealed that earlier during the month, the patient had returned from Rwanda, where infection was presumably acquired through protected heterosexual contact on the day (day 1) before departure to Germany (Figure 1).

Four days later, on day 5, the patient went to a neighbouring federal state for a private visit, but left on the same day to go back to North Rhine-Westphalia. On day 6, initial cutaneous lesions appeared in the genital area, while the patient had not experienced prodromal symptoms [4]. Five days post symptom-onset (day 11), the patient presented to the emergency department due to increasing pain and was hospitalised. During hospitalisation, symptoms progressed to widespread lesions affecting the face, body and genital area. After other infections were ruled out, the hospital staff collected swabs and a urine sample for mpox diagnostics on day 15 with results available the following day. All swabs were PCR positive for mpox with a high viral load of MPXV detected in the facial and genital swabs and lower viral load detected in the throat swab and urine. Upon these findings on day 16, the patient was immediately isolated. Given the patient’s travel history to Rwanda where mpox clade Ib cases had been confirmed [2], mpox clade Ib was suspected. This was confirmed on day 17 by typing results. During the hospital stay, the patient received medical supportive care but no specific antivirals. The patient had not previously been vaccinated against mpox or smallpox. From day 17 onwards, the patient developed no new lesions and recovered rapidly. By day 24, the patient only had a few remaining crusts which could be covered and was discharged from hospital. As a precautionary measure, a PCR test from the nose and throat was performed prior to their discharge, which was negative. Furthermore, the patient was instructed to wear a high filtration mask during their journey home, self-isolate for an additional 3 days at home until the last crusts had fallen off and to follow hygiene standards according to RKI recommendations [5,6].

## Public health actions

**Figure 2:**
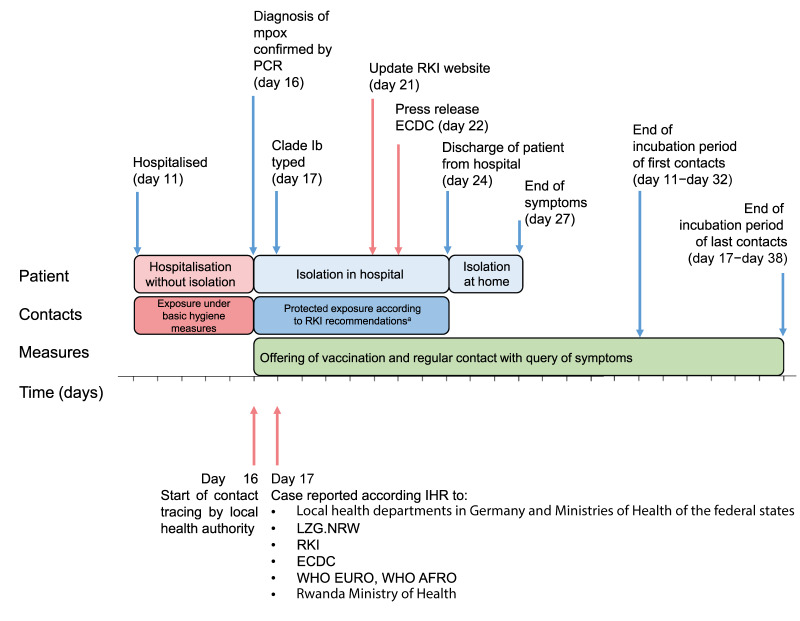
Timeline of public health responses after detection of first mpox clade Ib case in Germany, October 2024

ECDC: European Centre for Disease Prevention and Control; IHR: International Health Regulations; LZG.NRW: North Rhine-Westphalia Centre for Health (federal state public health institute); RKI: Robert Koch Institute (national public health institute in Germany); WHO: World Health Organization; WHO AFRO: WHO Regional Office for Africa; WHO EURO: WHO Regional Office for Europe.

^a^ Recommendations on hygiene measures for the treatment and care of patients with MPXV infection by RKI [5].

Upon high suspicion of a MPXV clade Ib infection in Germany, a wide public health response was implemented to prevent local transmission of mpox. This included isolating the case, contact tracing, providing guidance to the contacts, administering post-exposure prophylaxis, cross-institutional collaboration and rapid communication with the local, regional and national health departments in Germany (Figure 2).

From the moment the patient was isolated, all healthcare staff attending to the patient’s care wore full personal protective equipment (PPE), including gowns, gloves, eye protection and high filtration masks.

As foreseen in the German Infection Protection Act (IfSG) [7], the hospital notified the PCR-confirmed mpox diagnosis to the local health department of Cologne, which consulted with LZG.NRW and notified the suspicion of mpox clade Ib according to §12 IfSG to LZG.NRW on day 16. Staff of LZG.NRW reviewed the WHO 2022–2024 mpox outbreak dashboard, which showed that up to October 2024, only mpox clade Ib has been confirmed in Rwanda [2]. Using the RKI mpox flowchart, it was determined that there was a high probability that the mpox case was infected with  clade Ib MPXV so LZG.NRW informed RKI, the German national International Health Regulations (IHR) focal point for public health [8]. When the results confirmed that the case tested positive for clade Ib MPXV on day 17, LZG.NRW forwarded the notification of the local health department of Cologne to RKI. RKI conducted a risk assessment according to the IHR and informed the Rwanda Ministry of Health, the European Centre for Disease Prevention and Control (ECDC) and the WHO’s Regional Offices for Europe and Africa about the case [9]. The assessed risk for the German public, and for onwards international spread of the disease was considered low.

On day 21, the RKI updated their public webpage on mpox in Germany, which attracted broad media interest [10]. The Ministry of Labour, Health and Social Affairs of North-Rhine Westphalia reported on the case to the press upon request and a press release from ECDC followed on day 22 [11]. On day 23, the RKI informed the professional public about the mpox clade Ib case in Germany, providing details on the case demographics, exposure and risk assessment to prevent the transmission of mpox in Germany [3].

## Contact tracing

The local health department of Cologne started contact tracing immediately on day 16, adopting a highly sensitive contact definition that included all persons who were exposed to the patient while the patient had presented with symptoms. This also included hospital staff who were only exposed to the patient while wearing PPE, as well as any individual who entered the patient’s hospital room. The patient had no reported household or sexual contact since their arrival in Germany.

**Figure 3:**
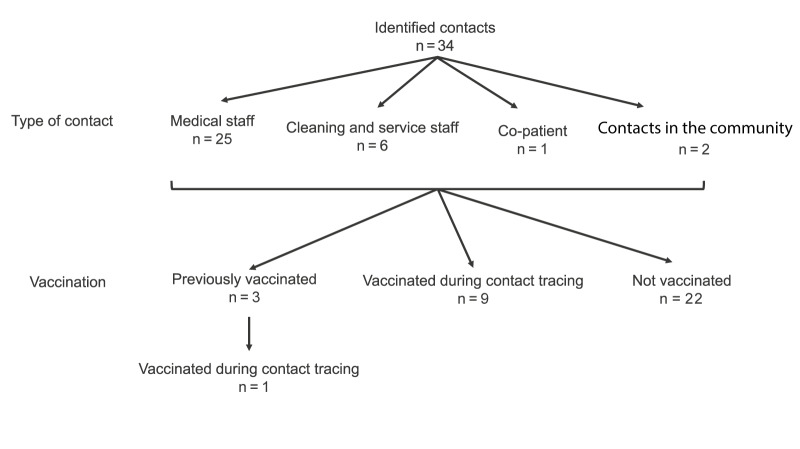
Contact tracing outcome with numbers of contacts followed-up for 21-days after their last exposure to the first mpox clade Ib case, as well as their vaccination status, Germany, October 2024 (n = 34 contacts identified)

Contact tracing identified a total of 34 contacts, all considered low risk, across six local health departments (five in North-Rhine Westphalia and one in a neighbouring German federal state), including two contacts in the community and 32 hospital contacts (physicians, nurses, cleaning and service staff, co-patient) (Figure 3). Contacts were followed for 21 days after their last exposure to the case, the maximum incubation period for mpox [4].

Status November 2024.

Of all 34 contacts, 22 reported close physical contact to the case, of whom 20 wore gloves, two wore mouth and nose protection and 18 disinfected their hands post-contact (Table). The time the contact person was exposed to the case was less than 1 min for half of the close contacts, and between 1 and 15 min for seven of the close contacts. For the 12 contacts who were not in direct contact to the case (e.g. shared a room, cleaned the room, replaced the food tray), only three reported prolonged exposure times of several hours or longer.

**Table:** Exposure details of contacts of the first mpox clade Ib case, Germany, October 2024 (n = 34 contacts)

Characteristics of contact	Number
**Reported close physical contact with the case^a^**	**22**
**Contact with protective measures (multiple choice possible)**	
Gloves worn	20
Mouth and nose protection	2
Hand disinfection	18
**Face-to-face interaction^b^**	
Face-to-face contact	17
No face-to-face contact	2
Unknown	3
**Exposure time**	
Less than 1 min	11
1–4 min	3
5–15 min	4
Several hours	0
Unknown	4
**Administration of post-exposure vaccination**	**10**
**Reported non-physical contact with the case^c^**	**12**
**Exposure time**	
Less than 1 min	4
1–4 min	1
5–15 min	0
Several hours	3
Unknown	1
None^d^	3

PPE: personal protective equipment.

^a^ Close physical contact was defined as contact involving direct touching.

^b^ Face-to-face interaction was defined as contact < 1 m.

^c^ Non-physical contact was defined as contact with no touching, including being present in the same room as the index case.

^d^ This refers mainly to staff who came in the case’s room to clean it in the absence of the case. They had no direct exposure to the case but could have been exposed to infectious material.

As most contacts were medical staff, nearly all of the 22 individuals who reported close contact to the mpox case wore gloves during contact and disinfected their hands post-contact. Without PPE, there would likely have been an increased probability for infection [12]. The two close community contacts who did not wear gloves did not come into direct contact with uncovered lesions. Additionally, the patient did not experience any respiratory symptoms, and the overall risk of transmission was therefore considered low. All contacts were advised according to current national German recommendations [5,6]. The responsible local health departments contacted all contacts regularly and asked them about the development of any symptoms, recording their responses and forwarding them to LZG.NRW. Additionally, all contacts were offered post-exposure vaccination. Of the 34 contacts, three were previously vaccinated against mpox or smallpox and 10 contacts chose to be vaccinated, including one who was previously vaccinated (Figure 3). The vaccine was administered from 1 to 8 days post exposure.

As all contacts were highly compliant and considered to be at low-risk of MPXV infection, no contacts were required to stay at home but were instructed to report any developing symptoms. Testing of asymptomatic contacts was not performed. During the 21-day observation period, six individuals developed unspecific symptoms (e.g. influenza-like infection, herpes zoster, pimples, headaches, allergic reactions), which were scrutinised by the public health department. There were no indications of mpox infection. As a precautionary measure, one of the six contact persons with unspecific symptoms was tested for mpox, and the test was negative. To ensure that the local health authorities were kept informed and provided with a platform for discussing any challenges, LZG.NRW organised weekly web conferences, held as needed throughout the 21-day observation period.

LZG.NRW supported that the public health measures of the six involved local health departments were as uniform and tailored as possible.

## Discussion

Mpox is an infectious disease caused by MPXV, an Orthopoxvirus, which manifests with influenza-like symptoms and a characteristic rash. It spreads through close contact with infected bodily fluids, sores, or scabs, including sexual contact [13]. MPXV strains are divided into clades with specific geographic distributions; human infections have been linked to clade I (and its two sub-lineages Ia and Ib) and clade II (and its two sub-lineages IIa and IIb) viruses [14]. A global mpox outbreak in 2022 was due to clade II, which is endemic in West Africa. During this outbreak, 3,959 cases were reported to the RKI in Germany of which 839 cases (21.2%) were reported to the LZG.NRW by local health departments in North Rhine-Westphalia [15]. After an increase of mpox clade II cases in Germany in May 2022, the number of cases fell significantly from August 2022 onwards, at least partially due to intensive public health efforts by various stakeholders. Since, only sporadic MPXV infections are reported in Germany.

In 2024, clade Ib, which was first detected in DRC, began to spread in some Central and East African countries including Rwanda [1,2]. Due to limited data, it remains unclear whether clade I and especially the sub-lineage Ib is inherently more transmissible or causes higher mortality than  clade II, although research suggests that  clade I is associated with more severe clinical symptoms [16,17]. Travel-associated imported mpox clade Ib cases outside of the African continent have since successively been confirmed in Sweden, Thailand, India, Germany and the United Kingdom [3,18-20]. While the overall risk of mpox for the general population in the European Union is considered low, imported mpox cases in travellers returning from Central and Eastern Africa are expected and public health preparedness is essential [21].

In this context, as soon as the imported mpox case in Germany was PCR confirmed, medical staff at the hospital where the case was admitted suspected mpox clade Ib infection, based on high awareness of the ongoing outbreak caused by MPXV of this sub-lineage in Africa. Appropriate prevention and control measures were immediately initiated. The management of the case was effective due to the high competence of the clinical and public health staff, who drew on experience from the 2022 MPVX clade II outbreak, for patient care, contact tracing and post-exposure prophylaxis. The German Permanent Working Group of Competence and Treatment Centres for High Consequence Infectious Diseases also provided treatment and clinical advice to the treating clinicians after the case was confirmed as infected with MPXV clade Ib. Additionally, staff at the involved public health institutes were well connected and experienced in notification and contact tracing protocols which facilitated rapid cross-institutional communication across local, regional, national and international levels. The organisation of frequent low-threshold meetings among involved stakeholders also made it possible to rapidly exchange information and keep stakeholders up to date.

Some limitations can be nevertheless mentioned. There was for example a delay in reaching the mpox diagnosis. As heterosexual transmission of MPXV has been rare in Germany and the patient’s first symptoms were restricted to genital lesions, clinicians initially suspected other pathogens, such as those causing common sexually transmitted infections. The delay in testing for MPXV extended the time the patient was hospitalised without isolation and therefore may have increased the risk for transmission.

## Conclusion

This report describes the management of the first laboratory-confirmed imported case of mpox clade Ib in Germany, highlighting the importance of a coordinated public health response to prevent disease transmission. As the mpox clade Ib outbreak in the African continent is ongoing, there is a continued risk of imported clade Ib cases into European countries in the future. Public health institutes should thus be prepared for managing imported cases by ensuring an established action framework, which outlines the rapid implementation of control measures upon case detection, the contact tracing process and cross-institutional communication pathways.

## References

[1] World Health Organization (WHO). Disease Outbreak News. Mpox - Democratic Republic of the Congo. Geneva: WHO, 2024. Available from: https://www.who.int/emergencies/disease-outbreak-news/item/2024-DON522

[2] World Health Organization (WHO). 2022-24 Mpox (Monkeypox) Outbreak: Global Trends. Geneva: WHO. [Accessed 7 Nov 2024]. Available from: https://worldhealthorg.shinyapps.io/mpx_global/

[3] Robert Koch Institute (RKI). Erster Nachweis einer Infektion mit Mpox Klade Ib. Epidemiologisches Bulletin; 2024, 43.RKI; 2024. Available from: https://www.rki.de/DE/Content/Infekt/EpidBull/Archiv/2024/Ausgaben/43_24.pdf?__blob=publicationFile

[4] European Centre for Disease Prevention and Control (ECDC). Factsheet for health professionals on mpox. Stockholm: ECDC; 2024. Available from: https://www.ecdc.europa.eu/en/all-topics-z/monkeypox/factsheet-health-professionals

[5] Robert Koch Institute (RKI). Recommendations of the Robert Koch Institute on Hygiene Measures for the Treatment and Care of Patients with Monkeypox Virus Infection in Healthcare Facilities. RKI; 2024. Available from: https://www.rki.de/DE/Content/InfAZ/A/Affenpocken/Hygiene.html

[6] Robert Koch Institute (RKI). Mpox (monkeypox) — Information for the public. RKI; 2024. Available from: https://www.rki.de/DE/Content/Infekt/EpidBull/Merkblaetter/Ratgeber_Mpox_Affenpocken.html

[7] Infektionsschutzgesetz (IfSG). [Infections Protection Act]. Bundesministerium der Justiz. German. [Accessed 28 Nov 2024]. Available from: https://www.gesetze-im-internet.de/ifsg/

[8] Robert Koch Institute (RKI). Mpox: Flow chart for suspicion clarification and measures, guidance for doctors. RKI; 2024. Available from: https://www.rki.de/DE/Content/InfAZ/A/Affenpocken/Flussschema.html

[9] World Health Organization (WHO). Strategic toolkit for assessing risks: a comprehensive toolkit for all-hazards health emergency risk assessment. Geneva: WHO; 2021. Licence: CC BY-NC-SA 3.0 IGO.

[10] Robert Koch Institute (RKI). Mpox in Germany. RKI; 2024. Available from: https://www.rki.de/DE/Content/InfAZ/A/Affenpocken/Ausbruch-2022-Situation-Deutschland.html

[11] European Centre for Disease Prevention and Control (ECDC). Confirmed mpox Clade Ib case in Germany, risk remains low for EU/EEA. Stockholm: ECDC; 2024. Available from: https://www.ecdc.europa.eu/en/news-events/confirmed-mpox- Clade-ib-case-germany-risk-remains-low-eueea

[12] Beeson A Styczynski A Hutson CL Whitehill F Angelo KM Minhaj FS Mpox respiratory transmission: the state of the evidence. Lancet Microbe. 2023;4(4):e277-83. 10.1016/S2666-5247(23)00034-4 36898398 PMC9991082

[13] Harris E . What to Know About Monkeypox. JAMA. 2022;327(23):2278-9. 10.1001/jama.2022.9499 35622356

[14] Ulaeto D Agafonov A Burchfield J Carter L Happi C Jakob R New nomenclature for mpox (monkeypox) and monkeypox virus clades. Lancet Infect Dis. 2023;23(3):273-5. 10.1016/S1473-3099(23)00055-5 36758567 PMC9901940

[15] Robert Koch Institute (RKI). Surveillance Statistics (Germany). SurvStat@RKI 2.0. RKI. [Accessed 11 Nov 2024]. Available from: https://survstat.rki.de

[16] Vakaniaki EH Kacita C Kinganda-Lusamaki E O’Toole Á Wawina-Bokalanga T Mukadi-Bamuleka D Sustained human outbreak of a new MPXV clade I lineage in eastern Democratic Republic of the Congo. Nat Med. 2024;30(10):2791-5. 10.1038/s41591-024-03130-3 38871006 PMC11485229

[17] Branda F Ceccarelli G Ciccozzi M Scarpa F . First cases of mpox Clade I outside of Africa: genetic insights on its evolution. Infect Dis (Lond). 2024;56(11):1003-5. 10.1080/23744235.2024.2399776 39260825

[18] Public Health Agency of Sweden (FOHM). One case of mpox Clade 1 reported in Sweden. Stockholm: FOHM; 2024. Available from: https://www.folkhalsomyndigheten.se/the-public-health-agency-of-sweden/communicable-disease-control/disease-information-about-mpox/one-case-of-mpox- Clade-i-reported-in-sweden

[19] Center for Infectious Disease Research and Policy (CIDRAP). More global mpox spread as Clade 1b confirmed in Thailand, the 2nd case outside Africa. Minneapolis: University of Minnesota; 2024. Available from: https://www.cidrap.umn.edu/mpox/more-global-mpox-spread- Clade-1b-confirmed-thailand-2nd-case-outside-africa

[20] United Kingdom Health Security Agency (UKHSA). Latest update on cases of Clade Ib mpox. London; UKHSA; 2024. Available from: https://www.gov.uk/government/news/ukhsa-detects-first-case-of- Clade-ib-mpox

[21] European Centre for Disease Prevention and Control (ECDC). Risk assessment for the EU/EEA of the mpox epidemic caused by monkeypox virus Clade I in affected African countries. ECDC; 2024. Available from: https://www.ecdc.europa.eu/en/news-events/confirmed-mpox- Clade-ib-case-germany-risk-remains-low-eueea

